# Contactin-1 Reduces E-Cadherin Expression Via Activating AKT in Lung Cancer

**DOI:** 10.1371/journal.pone.0065463

**Published:** 2013-05-28

**Authors:** Judy Yan, Nicholas Wong, Claudia Hung, Wendy Xin-Yi Chen, Damu Tang

**Affiliations:** 1 Division of Nephrology, Department of Medicine, McMaster University, Hamilton, Ontario, Canada; 2 Division of Urology, Department of Surgery, McMaster University, Hamilton, Ontario, Canada; 3 Father Sean O'Sullivan Research Institute, St. Joseph's Hospital, Hamilton, Ontario, Canada; 4 The Hamilton Center for Kidney Research, St. Joseph's Hospital, Hamilton, Ontario, Canada; Sun Yat-sen University Medical School, China

## Abstract

Contactin-1 has been shown to promote cancer metastasis. However, the underlying mechanisms remain unclear. We report here that knockdown of contactin-1 in A549 lung cancer cells reduced A549 cell invasion and the cell's ability to grow in soft agar without affecting cell proliferation. Reduction of contactin-1 resulted in upregulation of E-cadherin, consistent with E-cadherin being inhibitive of cancer cell invasion. In an effort to investigate the mechanisms whereby contactin-1 reduces E-cadherin expression, we observed that contactin-1 plays a role in AKT activation, as knockdown of contactin-1 attenuated AKT activation. Additionally, inhibition of AKT activation significantly enhanced E-cadherin expression, an observation that mimics the situation observed in contactin-1 knockdown, suggesting that activation of AKT plays a role in contactin-1-mediated downregulation of E-cadherin. In addition, we were able to show that knockdown of contactin-1 did not further reduce A549 cell's invasion ability, when AKT activation was inhibited by an AKT inhibitor. To further support our findings, we overexpressed CNTN-1 in two CNTN-1 null breast cancer cell lines expressing E-cadherin. Upon overexpression, CNTN-1 reduced E-cadherin levels in one cell line and increased AKT activation in the other. Furthermore, in our study of 63 primary lung cancers, we observed 65% of primary lung cancers being contactin-1 positive and in these carcinomas, 61% were E-cadherin negative. Collectively, we provide evidence that contactin-1 plays a role in the downregulation of E-cadherin in lung cancer and that AKT activation contributes to this process. In a study of mechanisms responsible for contactin-1 to activate AKT, we demonstrated that knockdown of CNTN-1 in A549 cells did not enhance PTEN expression but upregulated PHLPP2, a phosphatase that dephosphorylates AKT. These observations thus suggest that contactin-1 enhances AKT activation in part by preventing PHLPP2-mediated AKT dephosphrorylation.

## Introduction

The neural cell adhesion protein contactin-1 (CNTN-1) consists of six Ig domains, four fibronectin-like motifs, and a glycosylphosphatidylinositol (GPI)-moiety [1]. The GPI moiety anchors CNTN-1 to the external membrane surface of the central and peripheral neurons [2], [3]. CNTN-1 plays a role in axon extension and formation of septate-like junctions between axons and myelinating glial cells [4]–[6], In addition, CNTN-1 acts as a ligand to the Notch receptor in the brain resulting in oligodendrocyte maturation [7]. In line with these in vitro observations, in vivo studies reveal a critical role of CNTN-1 in axon guidance and synapse formation [8]–[10]. Knockdown of CNTN-1 in *Xenopus* embroys resulted in misguidance and the defasciculation of the trigeminal nerve axons [11]. Whereas, mice deficient in CNTN-1 die in a few weeks due to severe ataxia [4], [8].

Although the loss of CNTN-1 function, as a result of gene knockout or spontaneous mutations in *CNTN-1*, affects the central and peripheral nervous systems but not the neuromuscular junctions (NMJs) in mice [8], [12], mutations in the *CNTN-1* gene has been implicated to impair NMJs function in humans [13]. A mutation resulting in the introduction of a premature stop codon within the third Ig domain was associated with a familial form of lethal congenital myopathy in humans [13]. Despite the accumulating research on CNTN-1 function, little is known about its function outside of the nervous system. Although, northern blot analyses of pancreas, lung, kidney and skeletal muscle revealed only low levels of CNTN-1 transcripts [14], its function in these tissues and expression in other tissues has yet to be determined. Only recently have there been reports of CNTN-1 expression in diseases outside of the nervous system, most notably with its involvement in cancer. CNTN-1 was detected in primary lung cancer and knockdown of CNTN-1 in lung cancer cells specifically inhibited their metastasis but not the formation of local xenograft tumours in immunocompromised mice [15]. This is in part due to the essential role of CNTN-1 on actin cytoskeleton rearrangement and focal adhesion structures [15]. In addition, CNTN-1-mediated metastasis is regulated by VEGF-C and CNTN-1 enhances GTP-bound RhoA which is attributable to CNTN-1-promoted lung cancer invasion and metastasis [15], [16]. Lung cancer patients with high levels of CNTN-1 have poor prognosis [15]. Consistent with these reports, factors that enhances lung cancer metastasis also upregulates CNTN-1 [17]. Additionally, CNTN-1 has been reported in melanoma [18] and is associated with metastasis in gastric cancer, oral squamous cell carcinoma, and esophageal cancer [19]–[22].

Despite accumulating evidence supporting a role of CNTN-1 in cancer metastasis, the underlying mechanisms responsible for this process remains unclear. To further investigate CNTN-1-mediated oncogenesis, we have knocked-down CNTN-1 in A549 lung cancer cells. This led to an upregulation of E-cadherin. In primary lung carcinoma, high levels of CNTN-1 co-existed with low levels of E-cadherin. Mechanistically, CNTN-1 plays a role in AKT activation, which in turn inhibits E-cadherin expression.

## Materials and Methods

### Cell lines, plasmids and inhibitors

Lung cancer cell lines (A549 and H1299), breast cancer cell lines (MCF7, BT549, BT474, MDA-MB-453, T47D and ZR751), kidney cancer cell lines (A498 and 786-0), a cervical cancer cell line (HeLa), a glioblastoma cell line (U87) and 293T cells (human 293 kidney embryonic epithelial cells) were purchased from American Type Culture Collection (ATCC, Manassas, VA). A549, BT549, H1299, T47D, BT474, ZR751, MDA-MB-453 and 786-0 cells were cultured in RPMI 1640 media. MCF7, HeLa and 293T cells were cultured in DMEM and A498 and U87 cells were cultured in MEM media. All media were supplemented with 10% FBS (Sigma Aldrich, Oakville, ON) and 1% Penicillin-Streptomycin (Life Technologies, Burlington, ON). The identity of A549 was confirmed by STR analysis carried out by DDC Medical (Fairfield, OH). CNTN-1 shRNA was purchased from Santa Cruz Biotechnology (Santa Cruz, CA) and CNTN-1 isoform 3 cDNA was purchased from Open Biosystems (Huntsville, AL). The AKT inhibitor VIII was purchased from Calbiochem (EMD, Mississauga, ON). E-cadherin promoter driven luciferase construct was kindly provided by Dr. Antonio García de Herreros, Universitat Pompeu Fabra, Spain [23].

### Knockdown of CNTN-1

Hairpin shRNAs (control/Ctrl and CNTN-1) were expressed by a retroviral-based shRNA vector (Santa Cruz Biotechnology, Santa Cruz, CA). Knockdown of CNTN-1 was carried out according to our published conditions [24]–[26]. Briefly, a gag-pol expressing vector, a rev expressing vector and an envelope expressing vector (VSV-G) (Stratagene, Mississauga, ON) were transiently co-transfected with a designed retroviral plasmid into 293T cells. The virus-containing medium was harvested 48 hours later, filtered through a 0.45 µM filter, and centrifuged at 20,000 g for 120 minutes to concentrate the retrovirus. Polybrene (10 µg/ml, Sigma Aldrich, Oakville, ON) was added before infection and cells were selected for stable integration with puromycin (1 µg/mL, Sigma Aldrich, Oakville, ON).

### Retroviral overexpression of CNTN-1

Human CNTN-1 isoform 3 cDNA was purchased (Open Biosystems, Huntsville, AL) and further modified to generate the full length isoform 1 of CNTN-1. PCR primers were synthesized flanking the C terminus fragment present in isoform 1 but missing in isoform 3. RNA was isolated from A549 cells with TRIZOL (Life Technologies, Burlington, ON) and used as template for RT-PCR. The resulting C terminus PCR fragment was ligated into pBluescript KS+ and subsequently cut out at the restriction site for MfeI; a unique site present in isoform 1 and isoform 3, a few base pairs upstream before the two sequences differ. The C terminus fragment was then ligated with the commercially purchased isoform 3. The full length isoform 1 cDNA for CNTN-1 was subsequently cloned into a retroviral vector, pBabe. Confirmation of positive clones was determined by DNA sequencing. Overexpression of CNTN-1 was carried out using a gag-pol expressing vector and an envelope expressing vector (VSV-G) (Stratagene, Mississauga, ON). All steps were carried out in the same manner described above for the knockdown of CNTN-1. The pBabe vector without CNTN-1 was used as an empty vector control.

### Cell proliferation Assay

A total of 1000 cells of A549 shCTRL and shCNTN-1 cells was seeded into a 96 well plate and incubated at 37°C for 5 days. Proliferation was measured using the WST-1 cell proliferation assay kit (Millipore, Mississauga, ON) according to the manufacturer's instructions. Absorbance readings were measured with a plate reader at 420mn.

### Invasion assay

Modified boyden chambers were commercially purchased consisting of inserts with an 8 µm pore membrane coated with Matrigel (BD Biosciences, Mississauga, ON) placed in a 24-well plate. Invasion assays were performed according to the manufacturer's procedure. Briefly, matrigel inserts were given 2 hour to rehydrate at 37°C prior to use in the presence of 0.5 ml of medium. Complete medium (0.5 ml) supplemented with 10% fetal bovine serum (FBS) was placed in the lower chamber. A total of 5×10^4^ cells were seeded into the top chamber of the insert in 0.5 ml of serum-free medium for 22 hours. Cells that passed through the membranes were fixed and stained with crystal violet (0.5%, Sigma Aldrich, Oakville, ON). Percentage of invasive cells was calculated by dividing the number of cells passing through the 8 µm pore size matrigel membrane by the number of cells migrating through the control membrane and multiplying by 100.

### Anchorage-Independent Growth Assay

A549 shCTRL and A549 shCNTN cells were seeded into individual wells of six-well plates at a density of 10^4^ cells/well in 2mL of 2X media containing 0.25% agarose. After 3 weeks, 5 random fields per well were examined for colonies and counted under a phase-contrast microscope. Mean colony area was determined using Image Pro 5.0 software. Each experiment was conducted in triplicates and repeated three times.

### Western blot analysis

Cell lysates were prepared in a buffer containing 20 mM Tris (pH 7.4), 150 mM NaCl, 1 mM EDTA, 1 mM EGTA, 1% Triton X-100, 25 mM sodium pyrophosphate, 1 mM NaF, 1 mM β-glycerophosphate, 0.1 mM sodium orthovanadate, 1 mM PMSF, 2 µg/ml leupeptin and 10 µg/ml aprotinin (Sigma Aldrich, Oakville, ON). A total of 50 µg of cell lysate, unless otherwise specified was separated on SDS-PAGE gel and transferred onto Amersham hybond ECL nitrocellulose membranes (Amersham, Baie d'Urfe, QC). Membranes were blocked with 5% skim milk and then incubated with the indicated antibodies at 4°C overnight. Appropriate HRP-conjugated secondary antibodies were incubated for one hour at room temperature. Signals were detected using an ECL Western Blotting Kit (Amersham, Baie d'Urfe, QC). The primary and secondary antibodies and the concentrations used were: anti-CNTN-1 (1∶200, R&D Systems, Minneapolis, MN); anti-AKT (1∶1000, Santa Cruz Biotechnology, Santa Cruz, CA), anti-AKT Ser473 phosphorylation (1∶1000, Cell Signaling, Danvers, MA), anti-GSK3β Ser9 phosphorylation (1;1000, Cell Signaling, Danvers, MA), anti-GSK3β (1∶1000, Upstate Biotechnology, Billerica, MA), anti-E-cadherin (1∶2500, BD Biosciences, Mississauga, ON), anti-Snail (1∶200, Santa Cruz Biotechnology, Santa Cruz, CA), anti-PHLPP2 (1∶5000, Bethyl Laboratories, Montgomery, TX), anti-GAPDH (1∶5000, Cell Signaling, Danvers, MA), anti-actin (1∶1000, Santa Cruz), anti-goat (1∶3000, Santa Cruz Biotechnology, Santa Cruz, CA), anti-mouse (1∶3000, GE Healthcare, Mississauga, ON) and anti-rabbit (1∶3000, GE Healthcare, Mississauga, ON)

### Immunofluorescence staining

Cells were treated as defined in the figure legends. Immunofluorescence staining was carried out by fixing cells with 4% paraformaldehyde for 20 minutes and permeabilized with 0.05% saponin (Sigma Aldrich, Oakville, ON) for 15 minutes. Anti-CNTN-1 (1∶20, R&D systems, Minneapolis, MN) and anti-E-cadherin (1∶200, BD BioSciences, Mississauga, ON) were then added to the slides at 4°C overnight. After washing, secondary antibodies, FITC- or Rhodamine (TRITC) Donkey IgG (1∶200, Jackson ImmunoResearch Lab, West Grove, PA), were applied for 1 hour at room temperature. The slide was subsequently covered with VECTASHIELD mounting medium with DAPI (Vector Laboratories, Burlingam, CA). Images were taken with a fluorescence microscope (Carl Zeiss, Axiovert 200).

### Luciferase assay

A549 Ctrl shRNA and A549 CNTN-1 shRNA cells were co-transfected with pGL3 E-cadherin promoter-luciferase construct (kindly provided by Dr. Garcia de Herreros) and the pCH110-lacZ plasmid with Lipofectamine 2000 (Life Technologies, Burlington, ON). After 48 hours, luciferase (Promega, Madison, WI) and β-galactosidase activity was determined. Luciferase activity was normalized to β-galactosidase by dividing the luciferase activity signal with the β-galactosidase activity signal.

### Immunohistochemistry (IHC)

Tissue microarray (TMA) slides (LC723, LC10013) containing 63 lung adenocarcinomas were purchased from US Biomax (Rockville, MD). TMA slides were deparaffinized in xylene, cleared in ethanol series, and heat-treated for 30 minutes in sodium citrate buffer (pH = 6.0) in a food steamer. Primary antibodies specific for CNTN-1 (1∶50, R&D Systems, Minneapolis, NM) and E-cadherin (1∶400, BD Biosciences, Mississauga, ON) were incubated with the sections overnight at 4°C. Negative controls were incubated with a non-specific mouse, goat or rabbit IgG. Biotinylated secondary IgG and Vector ABC reagent (Vector Laboratories, Burlingam, CA) were subsequently added according to the manufacturer's instructions. Washes were performed with PBS. Chromogen reaction was carried out with diaminobenzidine (Vector Laboratories, Burlingam, CA), and counterstained with hematoxylin (Sigma Aldrich, Oakville, ON). TMA slides were scanned using a ScanScope and analyzed using ImageScope software (Aperio, Vista, CA). All spots (stained cores) were also manually examined. Scores obtained using the Imagescope software were converted to a HScore using the formula [(HScore  =  % positive X (intensity + 1)] [27], [28]. Scores were assigned to a scale of 0 to 3 (0 - negative or background staining, 1 - weak staining, 2 - modest staining, 3 - strong staining).

### Real time PCR analysis

Total RNA was isolated using TRIZOL (Life Technologies, Burlington, ON). Reverse transcription was carried out using superscript III (Life Technologies, Burlington, ON) according to the manufacturer's instruction. In brief, 2 µg of RNA was converted to cDNA at 65°C for 6 minutes followed by 2 minute incubation on ice, 25°C for 11 minutes, 50°C for 60 minutes and 70°C for 15 minutes. Real time PCR primers used for actin, PHLPP2, SIP1, Slug, Twist, E47 and E-cadherin are listed in Table 1. The PCR efficiency for each primer set is as follows: actin 93%, SIP1 86%, Slug 97%, E47 96%, Twist 92%, PHLPP2 92% and E-cadherin 85%. Real-time PCR was performed using the ABI 7500 Fast Real-Time PCR System (Applied Biosystems, Burlington, ON) in the presence of SYBR-green according to the manufacturer's instructions (Applied Biosystems, Burlington, ON). Briefly, each reaction consisted of 1 µL cDNA, 0.25 µL forward primer (10 µM), 0.25 µL reverse primer (10 µM), 4.75 µL H_2_O and 6.25 µL of SYBR green master mix. The PCR reaction was carried out in a 96 well plate at 50°C for 2 minutes, 95°C for 10 minutes, followed by 40 cycles at 95°C for 15 seconds and 60°C for 1 minute. All samples were run in triplicate and repeated three times.

**Table 1:** Real-time PCR primers.

Primers	Sequence
E-cadherin	CAAATCCAACAAAGACAAAGAAGGCAA ATGACAGACCCCTTAAAGACCTCCT
PHLPP2	AGGTTCCTGAGCATCTCT TC GTTCAGGCCCTTCAGTTGAG
Slug	TGACCTGTCTGCAAATGCTC CAGACCCTGGTTGCTTCAA
SIP1	CAATACCGTCATCCTCAGCA CCAATCCCAGGAGGAAAAAC
Twist	TCCATTTTCTCCTTCTCTGGAA GTCCGCGTCCCACTAGC
E47	CCCAGGAGCCTGAGCTG CTGTCACCAACGGGAAGG
Actin	ACCGAGCGCGGCTACAG CTTAATGTCACGCACGATTTCC

### Statistical analysis

Statistical analysis was performed using student t-test and p<0.05 was considered statistically significant.

## Results

### CNTN-1 reduces E-cadherin expression

**Figure 1:**
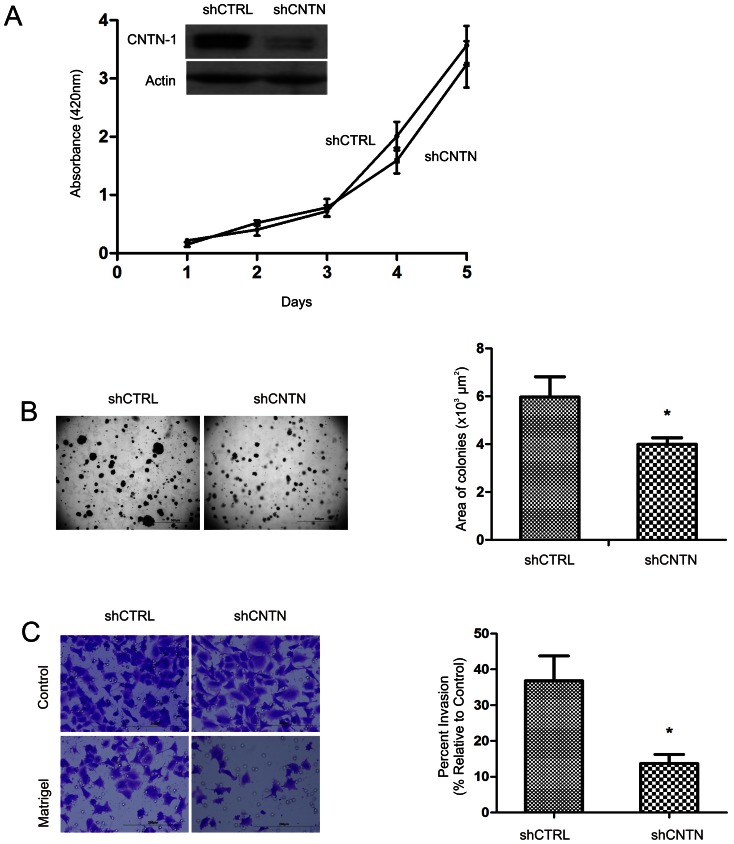
CNTN-1 promotes metastasis but not cell proliferation. (**A**) A549 cells were stably transfected with control (CTRL) or CNTN-1 shRNA. Knockdown of CNTN-1 were confirmed by western blot (inset). 1000 cells were seeded into 96 cell plates. Cell proliferation was assayed daily using WST-1 cell proliferation assay kit for five days. Experiments were repeated three times. Typical results from a single repeat are shown. (**B**) To examine the cell's ability to grow in soft agar, 10^4^ cells were seeded in agar containing medium for 3 weeks. Experiments were conducted in triplicates and repeated three times. Typical images from one experiment are shown (left panel). The sizes of soft agar colonies were calculated using ImagePro 5.0 software program and presented as means ± SD. *: *p*<0.05 in comparison to A549 shCNTN-1 cells (2 tailed student t-test). (**C**) A549 shCTRL and shCNTN were examined for their ability to pass through a control and matrigel membrane. Experiments were carried out three times. Typical images from one experiment are shown (left panel). Invasion was quantified (right panel).

CNTN-1 plays a critical role in the metastasis of A549 lung cancer cells [15]. To further investigate CNTN-1-mediated lung cancer metastasis, we have knocked-down CNTN-1 in A549 cells. While knockdown of CNTN-1 did not affect cell proliferation (Figure 1A), the cell's ability to grow in soft agar and to invade matrigel was significantly reduced (Figure 1B,C). These results are in line with the report that knockdown of CNTN-1 did not affect formation of xenograft tumors but reduced the cell's metastasis ability [15].

**Figure 2:**
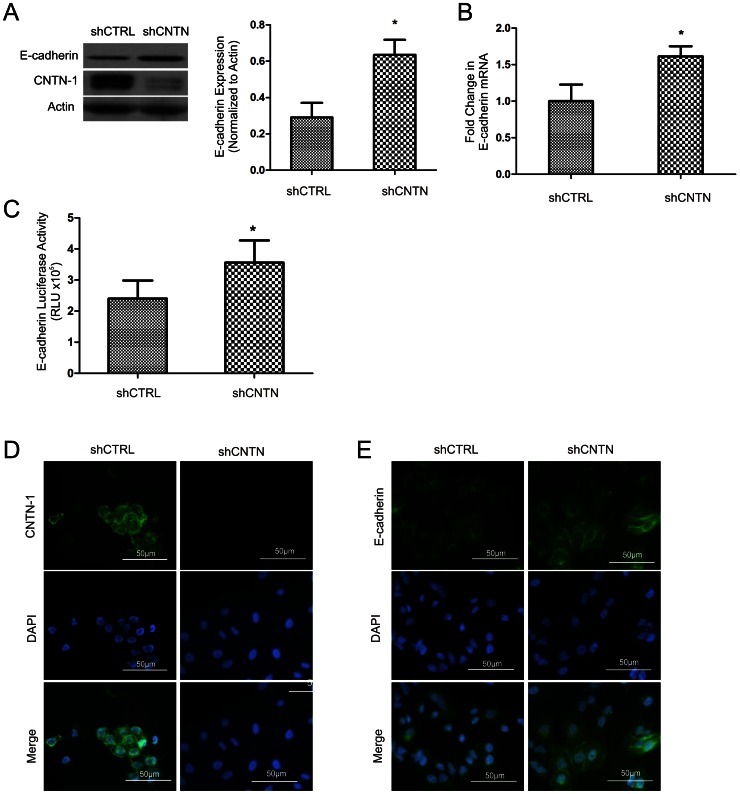
CNTN-1 reduces E-cadherin expression in A549 cells. (**A**) Cell lysates were prepared from the indicated cell lines, followed by the detection of E-cadherin, CNTN-1 and actin by western blot (left panel). Experiments were repeated three times. The levels of E-cadherin were quantified and graphed (means ± SD). *: *p*<0.05 by two-tailed student t-test. (**B**) Real time PCR analysis of E-cadherin expression in the indicated cell lines. β-actin was used as an internal control. E-cadherin mRNA in A549 shCNTN cells was shown as a fold change to that of A549 shCTRL cells. *: *p*<0.05 by two-tailed student t-test. (**C**) A549 shCTRL and A549 shCNTN cells were transiently transfected with E-cadherin promoter driven luciferase and a CMV driven LacZ construct for 48 hours, followed by assessing for luciferase and β-gal activities. Experiments were repeated three times. (**D**) Immunofluorescence staining of A549 shCTRL and A549 shCNTN for CNTN-1. (**E**) Immunofluorescence staining for E-cadherin on the indicated cell lines. Nuclei were counterstained with DAPI.

The invasion ability of epithelial cell-origin cancers is attributable to the loss of the epithelial cell adhesion protein, E-cadherin [29], [30]. To examine whether E-cadherin contributes to CNTN-1-medaited cell invasion, we were able to show that knockdown of CNTN-1 significantly increased E-cadherin expression (Figure 2A). This upregulation was in part due to the elevation in E-cadherin transcription, evidenced by the increase in E-cadherin mRNA (Figure 2B) and E-cadherin promoter activity (Figure 2C). Furthermore, consistent with CNTN-1 being anchored on the cell surface [3] and the site of function for E-cadherin also being at the cell surface, knockdown of CNTN-1 not only substantially reduced the cell surface content of CNTN-1 (Figure 2D) but also increased cell surface E-cadherin (Figure 2E). Taken together, the above observations demonstrate that CNTN-1 reduces E-cadherin expression at least in vitro.

**Figure 3:**
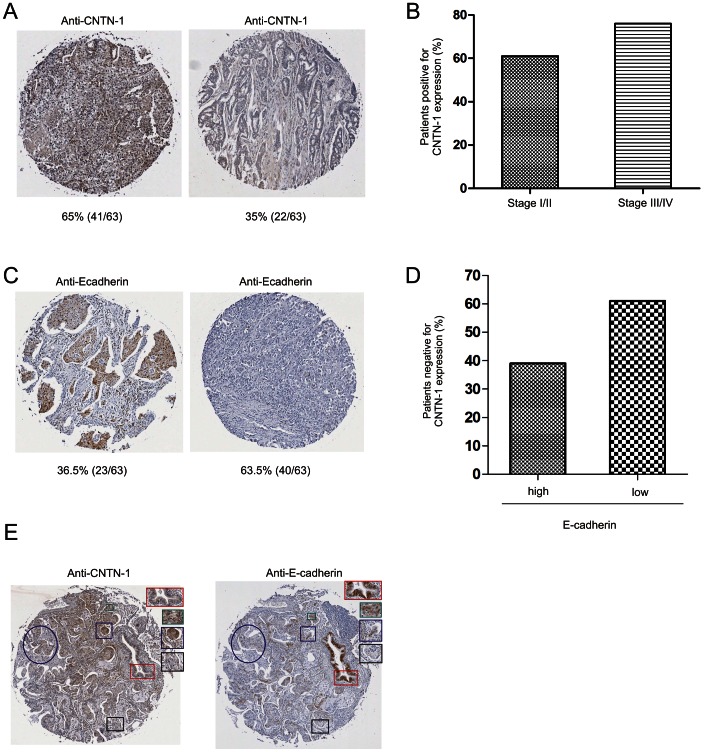
The association of CNTN-1 with E-cadherin in lung cancer progression. Sixty three primary lung carcinomas from tissue microarrays were IHC stained for CNTN-1 and E-cadherin. (**A**) Typical images of lung cancers with high and low levels of CNTN-1. The percentage of lung carcinomas with high or low levels of CNTN-1 is indicated. (**B**) Tissue microarrays were scanned and analyzed with ImageScope. CNTN-1 expression following lung cancer progression was analyzed. (**C**) Typical images of lung cancers with high and low levels of E-cadherin. The percentage of lung carcinomas with high or low levels of E-cadherin is indicated. (**D**) Based on IHC staining, the proportion of CNTN-1-positive carcinomas that expressed high or low levels of E-cadherin was calculated. (**E**) Primary lung cancer was IHC stained for CNTN-1 and E-cadherin. Regions positive for CNTN-1 and negative for E-cadherin (blue box, blue circle), positive for CNTN-1 and positive of E-cadherin (green box), negative for CNTN-1 and positive for E-cadherin (red box) and both CNTN-1 and E-cadherin negative (black box) can be observed.

To further determine the relationship between CNTN-1 and E-cadherin, we have examined 63 primary lung carcinomas (Table 2). Approximately 65% (41/63) and 35% (22/63) of primary lung carcinomas expressed readily detectable (CNTN-1^+^) and undetectable CNTN-1 (CNTN-1^−^), respectively by IHC (Figure 3A). This is consistent with the published incidence for CNTN-1^+^ versus CNTN-1^−^ primary lung carcinomas [15]. Additionally, in our analysis of 46 stages I/II and 17 stages III/IV primary lung carcinomas (Table 2), approximately 61% of stages I/II and 76% of stages III/IV carcinomas are CNTN-1-positive (Figure 3B), indicating a role of CNTN-1 in lung cancer progression.

**Table 2:** Patient information from TMA slides (US Biomax).

Patient ID	Sex	Age	Organ	Pathology	Grade	Stage	TMN
1	F	42	Lung	Adenocarcinoma	2	IIIb	T4N1M0
2	M	50	Lung	Adenocarcinoma	3	IIIa	T3N0M0
3	M	75	Lung	Adenocarcinoma	2	I	T2N0M0
4	F	59	Lung	Adenocarcinoma with necrosis	2	II	T2N1M0
5	M	62	Lung	Adenocarcinoma	2	IV	T2N1M1
6	M	51	Lung	Adenocarcinoma	2	II	T2N1M0
7	M	49	Lung	Adenocarcinoma	3	II	T1N1M0
8	M	59	Lung	Adenocarcinoma	2	II	T2N1M0
9	M	65	Lung	Adenocarcinoma	2	IIIa	T3N2M0
10	F	54	Lung	Adenocarcinoma	2	IIIa	T3N1M0
11	M	61	Lung	Adenocarcinoma with necrosis	2	II	T2N1M0
12	F	59	Lung	Adenocarcinoma with necrosis	2	I	T2N0M0
13	M	51	Lung	Adenocarcinoma	2	I	T1N0M0
14	F	37	Lung	Adenocarcinoma	2	I	T2N0M0
15	F	52	Lung	Adenocarcinoma	2	II	T2N1M0
16	F	52	Lung	Adenocarcinoma	2	II	T2N1M0
17	M	62	Lung	Adenocarcinoma	2	I	T2N0M0
18	F	49	Lung	Adenocarcinoma	2	I	T2N0M0
19	F	42	Lung	Adenocarcinoma	2	I	T2N0M0
20	M	55	Lung	Adenocarcinoma	2	I	T2N0M0
21	M	70	Lung	Adenocarcinoma	2	I	T2N0M0
22	M	52	Lung	Adenocarcinoma	2	II	T2N1M0
23	F	37	Lung	Adenocarcinoma	2	I	T2N0M0
24	F	63	Lung	Adenocarcinoma	2	I	T2N0M0
25	F	64	Lung	Adenocarcinoma	2	I	T2N0M0
26	M	70	Lung	Adenocarcinoma (lung tissue)	-	I	T2N0M0
27	F	62	Lung	Adenocarcinoma	2	I	T2N0M0
28	M	64	Lung	Adenocarcinoma	3	I	T2N0M0
29	M	63	Lung	Adenocarcinoma	3	I	T2N0M0
30	M	58	Lung	Adenocarcinoma	2	I	T2N0M0
31	F	32	Lung	Adenocarcinoma	3	I	T2N0M0
32	M	69	Lung	Adenocarcinoma	3	I	T2N0M0
33	F	68	Lung	Adenocarcinoma	3	II	T2N1M0
34	F	61	Lung	Adenocarcinoma (carcinoma sparse)	-	I	T2N0M0
35	M	62	Lung	Adenocarcinoma	3	IIIa	T3N0M0
36	F	72	Lung	Adenocarcinoma	3	II	T2N1M0
37	M	60	Lung	Adenocarcinoma	3	I	T2N0M0
38	M	49	Lung	Adenocarcinoma	3	I	T2N0M0
39	M	65	Lung	Adenocarcinoma	3	IIIa	T3N0M0
40	F	36	Lung	Adenocarcinoma (fibrous tissue and blood vessel)	-	IIIa	T2N2M0
41	M	39	Lung	Adenocarcinoma	3	I	T2N0M0
42	F	58	Lung	Adenocarcinoma	-	IIIa	T3N0M0
43	M	42	Lung	Adenocarcinoma	2	II	T2N1M0
44	M	70	Lung	Adenocarcinoma	2	I	T2N0M0
45	F	54	Lung	Adenocarcinoma	2	II	T2N1M0
46	M	61	Lung	Adenocarcinoma	2	II	T2N1M0
47	M	55	Lung	Adenocarcinoma	2	II	T2N1M0
48	F	63	Lung	Adenocarcinoma	2	II	T2N1M0
49	M	68	Lung	Adenocarcinoma (sparse)	2	IIIa	T3N0M0
50	M	49	Lung	Adenocarcinoma	2	IIIa	T3N0M0
51	M	58	Lung	Adenocarcinoma	2	II	T2N1M0
52	M	69	Lung	Adenocarcinoma	2	IIIa	T3N1M0
53	F	59	Lung	Adenocarcinoma	2	IIIa	T3N1M0
54	M	54	Lung	Adenocarcinoma	2	IIIa	T3N1M0
55	F	61	Lung	Adenocarcinoma	2	I	T2N0M0
56	F	58	Lung	Adenocarcinoma	2	IIIa	T3N1M0
57	M	51	Lung	Adenocarcinoma	2	IIIa	T3N1M0
58	M	70	Lung	Papillary adenocarcinoma	2	I	T2N0M0
59	F	56	Lung	Adenocarcinoma	2	I	T2N0M0
60	M	49	Lung	Adenocarcinoma	2	II	T2N1M0
61	M	47	Lung	Adenocarcinoma	2	IIIb	T4N0M0
62	M	53	Lung	Adenocarcinoma (sparse)	2	I	T2N0M0
63	F	50	Lung	Adenocarcinoma	2	I	T1N0M0

We also analyzed E-cadherin expression in primary lung carcinoma. E-cadherin^+^ and E-cadherin^−^ carcinomas were observed (Figure 3C) with the majority of cases being E-cadherin-negative (63% or 40/63). This is in line with a number of publications demonstrating 60%-70% of lung adenocarcinoma expressing reduced E-cadherin expression [31], [32]. However, others have also reported lower percentages, less than 50% of lung cancers expressing reduced E-cadherin [33], [34]. Importantly, approximately 61% of CNTN-1 positive carcinomas are also E-cadherin-negative (Figure 3D). However, we did observe carcinomas that were negative for both CNTN-1 and E-cadherin (data not shown), suggesting that CNTN-1 is not the only factor inhibiting E-cadherin expression. In supporting this suggestion, while CNTN-1 negative lung cancer regions could be E-cadherin positive, from the same patient the CNTN-1 positive lung carcinomas expression reduced levels of E-cadherin (Figure 3E). Taken together, our investigation supports the concept that CNTN-1 facilitates lung cancer progression/metastasis in part via downregulation of E-cadherin.

### CNTN-1 decreases E-cadherin expression via enhancing AKT activation

**Figure 4:**
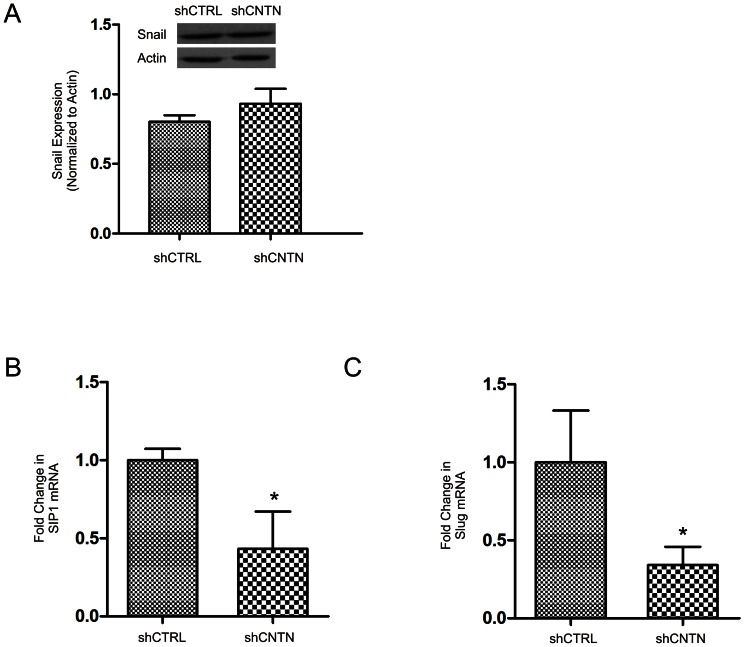
CNTN-1 mediated upregulation of E-cadherin is not due to Snail, but the result of Slug and SIP1. (**A**) Cell lysates for the indicated cell lines was examined for snail expression by western blot. Experiments were performed three times. Typical images (inset) and quantification of Snail expression are shown. Real time PCR analysis of (**B**) SIP1 and (**C**) Slug expression on the indicated cell lines. β-actin was used as an internal control. The mRNA in A549 shCNTN cells was shown as a fold change to that of A549 shCTRL cells. *: *p*<0.05 by two-tailed student t-test.

To examine the mechanisms responsible for CNTN-1-mediated downregulation of E-cadherin expression, we first determined whether CNTN-1 affects snail expression. Snail is the most widely studied inhibitor of E-cadherin transcription [23]. In A549 cells, knockdown of CNTN-1 does not change snail expression (Figure 4A), suggesting that snail may not be the major factor involved in CNTN-1-mediated inhibition of E-cadherin expression in A549 cells. Upon examination of other E-cadherin transcription factors, SIP1 and Slug expression decreased after CNTN-1 knockdown in A549 cells (Figure 4B, C). However, no change was seen for E47 and Twist (data not shown).

**Figure 5:**
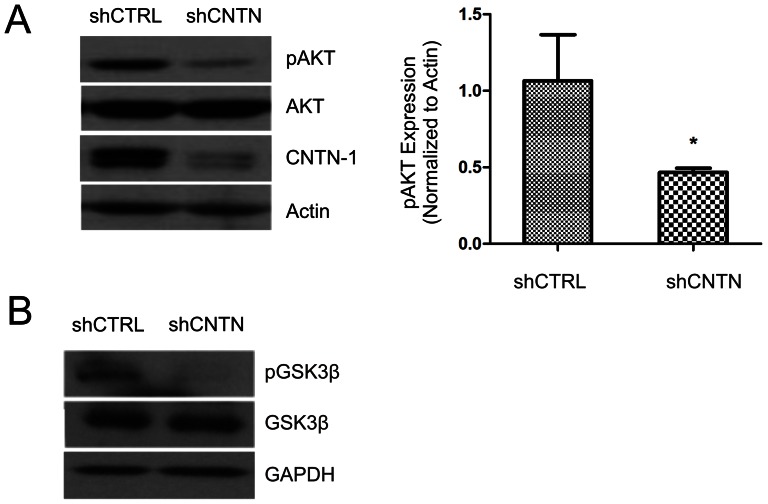
CNTN-1 plays a role in AKT activation during lung cancer tumorigenesis. (**A**) Cell lysates for A549 shCTRL and A549 shCNTN was examined for p-AKT and total AKT by western blot (left panel). AKT activation was quantified (right panel). (**B**) Phosphorylation at Ser9 of GSK3β (pGSK3β), GSK3β, and GAPDH expression in A549 shCTRL and A549 shCNTN cells were also determined.

Others and we have recently shown that AKT activity reduces E-cadherin expression [35]–[39] and AKT activity plays a critical role in tumorigenesis and metastasis [40]–[42]. We have thus examined whether AKT contributes to CNTN-1-mediated downregulation of E-cadherin. To investigate this possibility, we determined the status of AKT activation in A549 control cells and in A549 cells in which CNTN-1 was knocked-down. In comparison to shCTRL cells, knockdown of CNTN-1 significantly reduced AKT activation (Figure 5A). To further confirm changes in AKT activation, we demonstrated that in comparison to shCTRL cells phosphorylation of serine 9 of GSK3β, a well established AKT target [41], was significantly reduced in CNTN-1 knockdown cells (Figure 5B). Taken together, these observations reveal that CNTN-1 plays a role in AKT activation.

**Figure 6:**
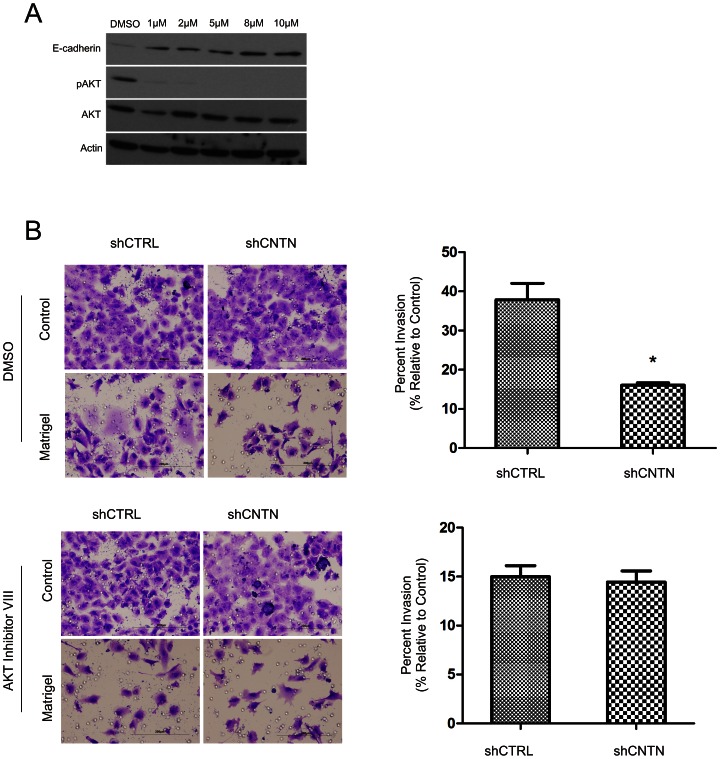
CNTN-1 reduces E-cadherin expression via AKT activation. (**A**) A549 cells were treated with an AKT inhibitor (AKT inhibitor VIII) at increasing concentrations and then examined for E-cadherin, AKT activation (pAKT), AKT, and Actin expression. (**B**) A549 shCTRL and A549 shCNTN-1 cells were mock-treated (DMSO, top two panels) or treated with an AKT inhibitor (bottom two panels), followed by determining their invasion capacity matrigel inserts. Experiments were repeated three times. Both typical images and quantification of cell's invasion ability are shown. *: *p*<0.05 by two-tailed student t-test.

We then determined whether modulation of AKT activity contributes to CNTN-1-induced decrease of E-cadherin expression. Inhibition of AKT activation with an AKT inhibitor increased E-cadherin expression in A549 cells (Figure 6A), indicating that reduction of AKT activation upon knockdown of CNTN-1 may contribute to the observed inhibition of A549 cell invasion (Figure 1). To test this possibility, we were able to show that while knockdown of CNTN-1 reduced A549 cell invasion upon DMSO treatment (vesicle control), knockdown of CNTN-1 did not further inhibit A549 cell invasion when AKT activation was inhibited (Figure 6B). Taken together, these observations support the notion that CNTN-1 inhibits E-cadherin expression via enhancing AKT activation. As reduction in E-cadherin plays a vital role in cancer metastasis [29], [30], loss of E-cadherin therefore contributes to lung cancer metastasis.

### CNTN-1 increases AKT activation by reducing PHLPP2 expression

**Figure 7:**
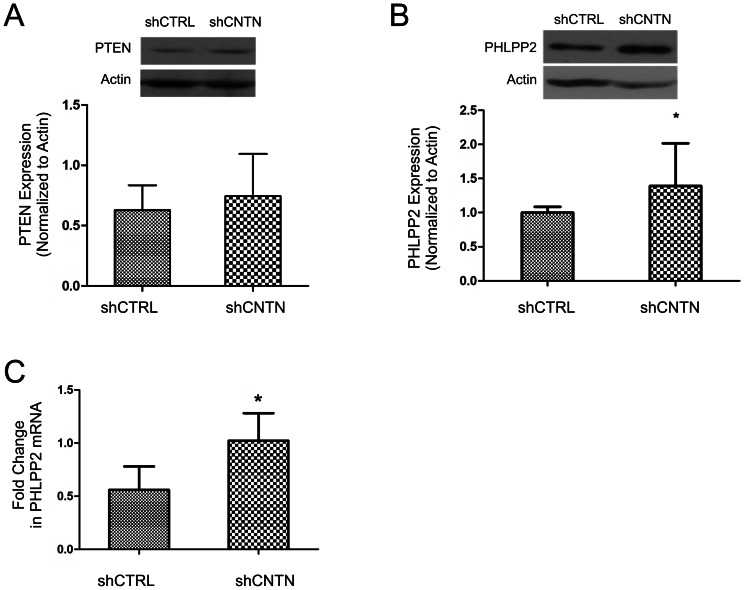
CNTN-1 activates AKT by downregulating PHLPP2. (**A**) A549 shCTRL and A549 shCNTN-1 cell lysates were examined for PTEN expression by western blot (top). Experiments were performed three times. Typical images from a single experiment were shown (left panel). PTEN expression was also quantified (right panel). (**B**) PHLPP2 expression in A549 shCTRL and A549 shCNTN cell lines were examined by western blot (top). Experiments were repeated three times. Typical images from a single experiment were shown (left panel). PHLPP2 expression was quantified (right panel). *: *p*<0.05 by two-tailed student t-test. (**C**) Real time PCR analysis of PHLPP2 expression in the indicated cell lines. β-actin was used as an internal control.

AKT activity is regulated by both upstream and downstream phosphatases, PTEN and PHLPP (PH domain leucine-rich repeat protein phosphatase). We therefore determined whether either or both phosphatases are involved in CNTN-1 knockdown-induced reduction of AKT activation. In comparison to shCTRL cells, knockdown of CNTN-1 did not significantly affect PTEN expression (Figure 7A). However, reduction in CNTN-1 significantly increased PHLPP2 expression in A549 cells (Figure 7B). Furthermore, upregulation of PHLPP2 in CNTN-1 knockdown A549 cells was in part attributable to the increase in PHLPP2 mRNA (Figure 7C), which may be the result of either upregulation of PHLPP2 gene transcription or stabilization of PHLPP2 mRNA.

### CNTN-1 regulation of E-cadherin and AKT activation is not unique to A549

**Figure 8:**
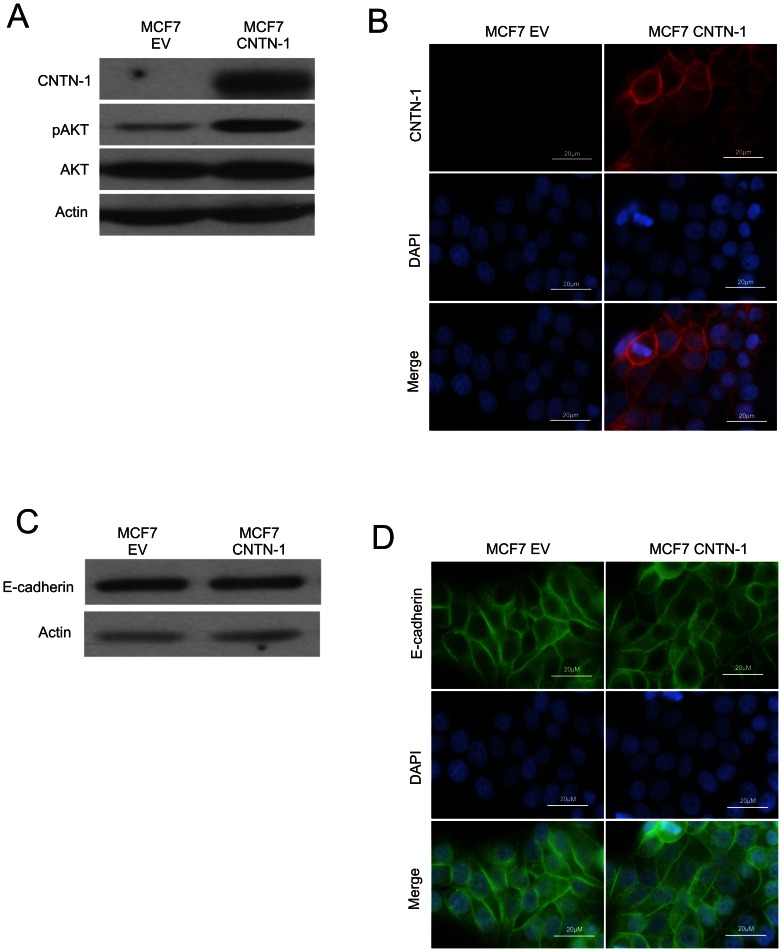
Overexpression of CNTN-1 activates AKT activity in MCF7 cells. (**A**) CNTN-1 was overexpressed in MCF7 cells and cell lysates were collected and run on western blot for CNTN-1, p-AKT, AKT and Actin expression. (**B**) Immunofluorescence staining for CNTN-1 on the indicated cell lines. (**C**) Cell lysates were collected from the indicated cell lines. Only 10 µg of cell lysates was run on western blot for E-cadherin and actin expression. (**D**) Immunofluorescence staining for E-cadherin on the indicated cell lines. Nuclei were counterstained with DAPI.

To determine if CNTN-1 regulates E-cadherin and AKT in other cancer cell lines, we examined a number of breast, kidney, lung and cervical cancers for CNTN-1 and E-cadherin expression. Despite the wide variety of cancers examined, CNTN-1 is not a universally expressed protein in cancer (Figure S1). In addition, since two breast cancer cell lines examined expressed E-cadherin, we proceeded to examine if CNTN-1 can regulated E-cadherin and AKT activity in these two cell lines. Upon ectopic overexpression of CNTN-1 in BT549, we observed a decrease in E-cadherin expression compared to empty vector control with no change in AKT activation (Figure S2). In contrast, overexpression of CNTN-1 in MCF7 cells led to an increase in AKT activation compared to empty vector control (Figure 8A,B), however, there was no change observed in E-cadherin (Figure 8C,D). Based on these evidences, CNTN-1 mediated regulation of E-cadherin and AKT is not restricted to lung cancer and may play a role in other cancers expressing CNTN-1.

## Discussion

CNTN-1 is a neural adhesion protein with functions in axon guidance and synapse formation [8]–[10]. It is intriguing that cancer cells may have explored these properties for metastasis. But how CNTN-1 promotes tumorigenesis remains incompletely understood. We demonstrated here that one of the unknown mechanisms is inhibiting E-cadherin expression. This concept is based on our study of the knockdown of CNTN-1 in A459 cells as well as the examination of CNTN-1 and E-cadherin in 63 primary lung carcinomas. As A459 cells already express a high level of CNTN-1 [15], we did not attempt to overexpress it in these cells. Nonetheless, our research is consistent with publications showing that CNTN-1 promotes lung cancer metastasis [15], [16] and that E-cadherin is a major contributor to the invasion and metastasis of epithelium-origin cancers [30], [31]. However, CNTN-1 is not the sole factor that inhibits E-cadherin expression, which is in line with the well established notion that E-cadherin is inhibited by multiple factors, including Snail, Twist, ZEB1, SIP1, and E12/E47 [43], [44].

Consistent with the reduction of E-cadherin which is mainly achieved through transcription inhibition [43], [44], knockdown of CNTN-1 enhanced E-cadherin transcription. As CNTN-1 is a cell surface protein (Figure 2D), CNTN-1 may indirectly inhibit E-cadherin expression through E-cadherin transcription inhibitors. Although Snail, E47 and Twist are not involved in this process, we identified a decrease in SIP1 and Slug which may attribute to the CNTN-1 mediated reduction of E-cadherin. Despite CNTN-1 playing a role in inhibiting E-cadherin expression at the transcriptional level, we cannot exclude the possibility that CNTN-1 may also downregulate E-cadherin at the protein level.

While the mechanisms whereby CNTN-1 reduces E-cadherin expression needs further investigation, we provide evidence that CNTN-1 decreases E-cadherin expression possibly by activating AKT. This conclusion is based on the observations that 1) knockdown of CNTN-1 reduced AKT activation in A549 cells, 2) inhibition of AKT activation robustly upregulated E-cadherin expression, and 3) knockdown of CNTN-1 was without effects on A549 cells invasion when AKT activity was inhibited. Additionally, we further provided evidence on CNTN-1 mediated effects on AKT and E-cadherin as the reverse was observed when CNTN-1 was overexpressed in MCF7 and BT549, respectively. Although we observed an increase in AKT activation in MCF7 cells upon ectopic overexpression of CNTN-1, there was no difference in E-cadherin levels. This can be attributed to the extremely high endogenous levels of E-cadherin in MCF7 cells. As little as 10 µg of protein was enough to provide a strong signal with western blotting (Figure 8). As a result, the high levels of endogenous E-cadherin in MCF7 cells may have masked any changes in E-cadherin levels upon ectopic overexpression of CNTN-1. In addition, although AKT activation increased after the overexpression of CNTN-1 in MCF7, there was no change in another breast cancer cell line, BT549 despite a change in E-cadherin levels (Figure S2). However, as oppose to MCF7 cells with low AKT activity due to its positive PTEN status, BT549 which is negative for PTEN shows high levels of endogenous AKT activity [45], [46]. This high level of endogenous AKT activity may explain why overexpression of CNTN-1 did not affect AKT activity in BT549.

How CNTN-1 activates AKT requires further investigation. Our research suggests that inhibition of PHLPP2 instead of PTEN contributes to CNTN-1-facilitated AKT activation. In the neuronal system, CNTN-1 has been shown to bind protein phosphatases, including protein tyrosine phosphatases ζ/β, PTPRZ, as well as PTPRG, and these interactions have functional consequences [47], [48]. Therefore, it will be interesting to examine whether CNTN-1 binds to protein phosphatase PHLPP2 and whether this interaction results in the inhibition of PHLPP2's phosphatase activity towards AKT. CNTN-1 contains six Ig domains, four fibronectin-like motifs, and a glycosyl phosphatidylinositol (GPI)-moiety [1]. The second and third Ig repeats interact with PTPRZ and PTPRG [47]. Therefore, it may be of interest to determine the structural elements of CNTN-1 that may be critical in its tumorigenic functions.

Despite the lack of research in the regulation of PHLPP2, PHLPP1 has recently been shown to be negatively regulated by AKT. GSK-3β can phosphorylate PHLPP1 leading to its ubiquitination and subsequent degradation via β-TrCP [49]. However, phosphorylation of GSK-3β by AKT inhibits this activity and thus directly influencing the stability of PHLPP1, resulting in a negative feedback loop to control AKT activation [49]. Dysregulation in this negative feedback loop was reported in a subset of high grade glioblastomas, where the level of active AKT determining the expression of its negative regulator PHLPP1 is lost [50]. This is due to the localization of β-TrCP1 to the nucleus as oppose to cytoplasm leading to a dysregulation of PHLPP1 levels [50]. Interestingly, a majority of glioblastoma cell lines tested also revealed reduced levels of PHLPP1 mRNA [50]. Whether PHLPP2 is regulated in a similar manner as PHLPP1 with dysregulations in its expression in lung cancer can be examined in the future.

## Supporting Information

Figure S1 **Expression of CNTN-1 and E-cadherin in various cancer cell lines.** Cell lysates were prepared from the indicated cell lines, followed by detection of CNTN-1, E-cadherin and actin by western blot.
(TIF)

Figure S2 **Overexpression of CNTN-1 decreases E-cadherin expression in BT549.** Cell lysates were collected for the indicated cell lines and run on western blot for E-cadherin, CNTN-1, p-AKT, AKT and actin.
(TIF)
