# Supplementary figures and images for: Contactin-1 Reduces E-Cadherin Expression Via Activating AKT in Lung Cancer
Source: PLoS One. 2013 May 28;8(5):e65463. doi: 10.1371/journal.pone.0065463 (PMC3665745; doi:10.1371/journal.pone.0065463)

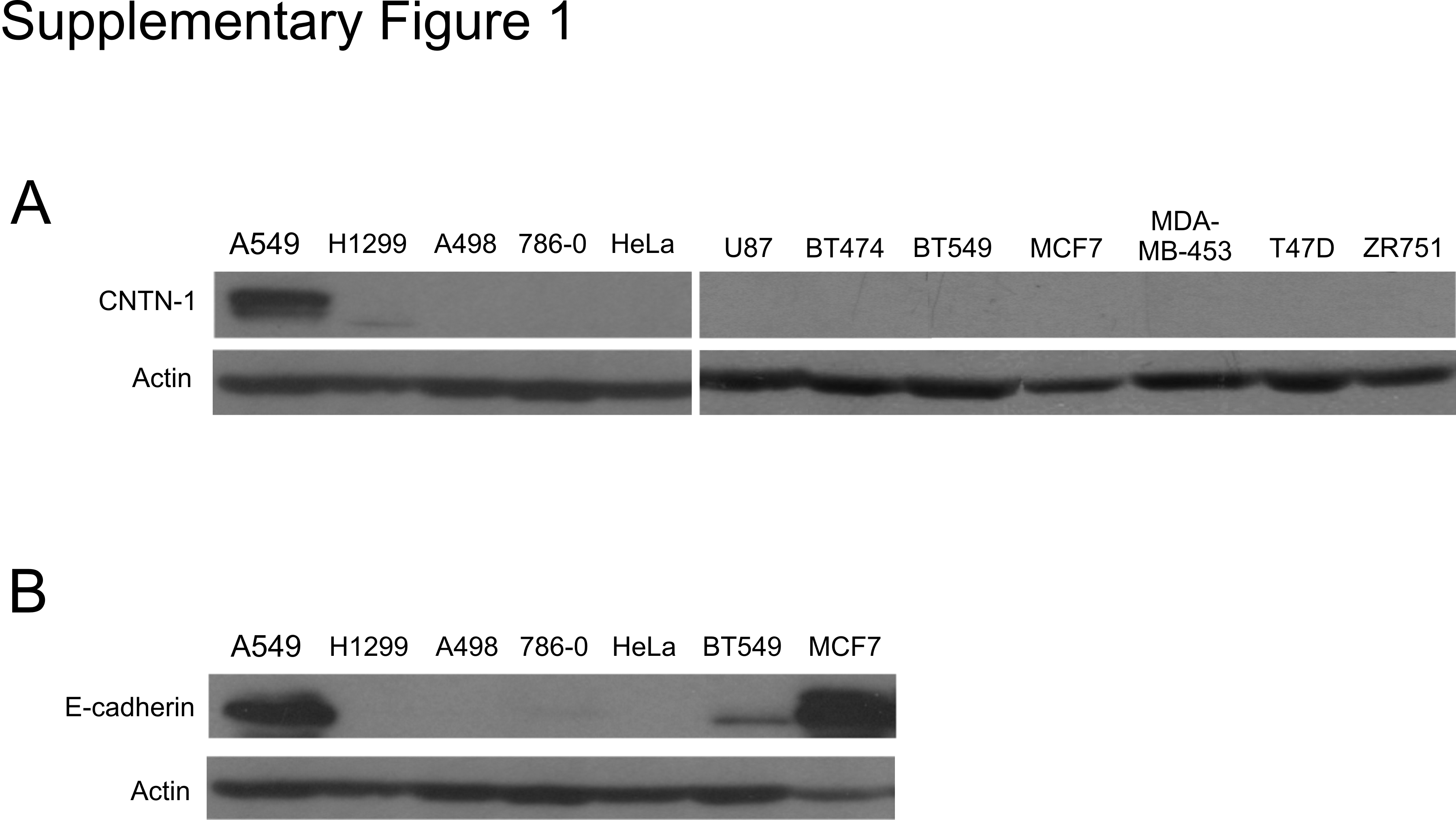

Supplement: Figure S1 — Expression of CNTN-1 and E-cadherin in various cancer cell lines. Cell lysates were prepared from the indicated cell lines, followed by detection of CNTN-1, E-cadherin and actin by western blot. (TIF) [file pone.0065463.s001.tif]

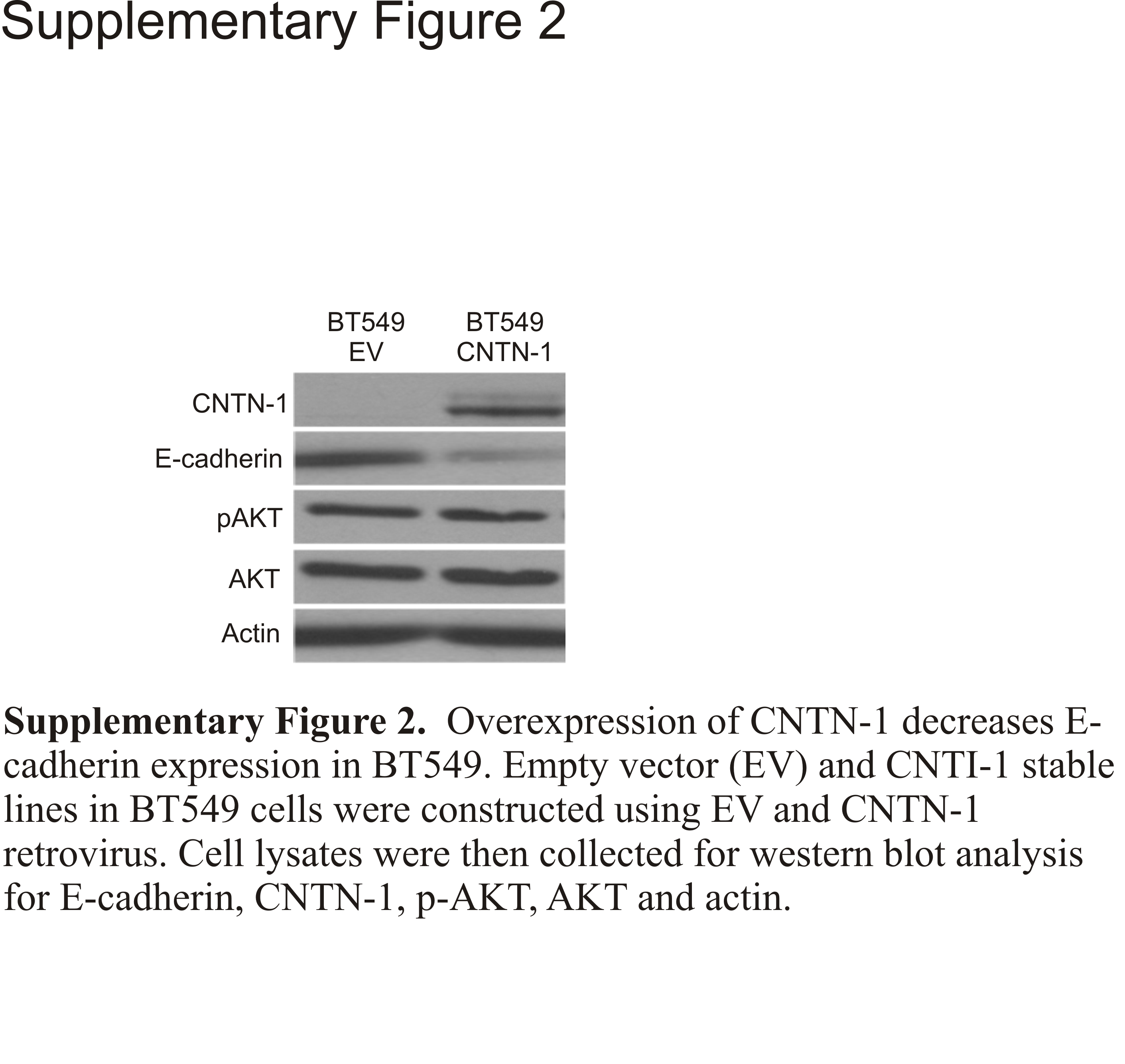

Supplement: Figure S2 — Overexpression of CNTN-1 decreases E-cadherin expression in BT549. Cell lysates were collected for the indicated cell lines and run on western blot for E-cadherin, CNTN-1, p-AKT, AKT and actin. (TIF) [file pone.0065463.s002.tif]
